# The Compromised Fanconi Anemia Pathway in Prelamin A‐Expressing Cells Contributes to Replication Stress‐Induced Genomic Instability

**DOI:** 10.1002/advs.202307751

**Published:** 2024-06-18

**Authors:** Pengqing Nie, Cheng Zhang, Fengyi Wu, Shi Chen, Lianrong Wang

**Affiliations:** ^1^ Department of Gastroenterology Hubei Clinical Center and Key Laboratory of Intestinal and Colorectal Disease Ministry of Education Key Laboratory of Combinatorial Biosynthesis and Drug Discovery Taikang Center for Life and Medical Sciences Zhongnan Hospital of Wuhan University, School of Pharmaceutical Sciences, Wuhan University Wuhan 430071 China; ^2^ Department of Infectious Diseases Institute of Pediatrics Shenzhen Children's Hospital Shenzhen Guangdong 518038 China; ^3^ Department of Burn and Plastic Surgery Shenzhen Institute of Translational Medicine, Medical Innovation Technology Transformation Center of Shenzhen Second People's Hospital Shenzhen Key Laboratory of Microbiology in Genomic Modification & Editing and Application Guangdong Provincial Key Laboratory of Systems Biology and Synthetic Biology for Urogenital Tumors Shenzhen University Medical School, Shenzhen Second People's Hospital, The First Affiliated Hospital of Shenzhen University Shenzhen 518035 China

**Keywords:** Fanconi anemia pathway, prelamin A, replication stress, senescence

## Abstract

Genomic instability is not only a hallmark of senescent cells but also a key factor driving cellular senescence, and replication stress is the main source of genomic instability. Defective prelamin A processing caused by lamin A/C (*LMNA*) or zinc metallopeptidase STE24 (*ZMPSTE24*) gene mutations results in premature aging. Although previous studies have shown that dysregulated lamin A interferes with DNA replication and causes replication stress, the relationship between lamin A dysfunction and replication stress remains largely unknown. Here, an increase in baseline replication stress and genomic instability is found in prelamin A‐expressing cells. Moreover, prelamin A confers hypersensitivity of cells to exogenous replication stress, resulting in decreased cell survival and exacerbated genomic instability. These effects occur because prelamin A promotes MRE11‐mediated resection of stalled replication forks. Fanconi anemia (FA) proteins, which play important roles in replication fork maintenance, are downregulated by prelamin A in a retinoblastoma (RB)/E2F‐dependent manner. Additionally, prelamin A inhibits the activation of the FA pathway upon replication stress. More importantly, FA pathway downregulation is an upstream event of p53‐p21 axis activation during the induction of prelamin A expression. Overall, these findings highlight the critical role of FA pathway dysfunction in driving replication stress‐induced genomic instability and cellular senescence in prelamin A‐expressing cells.

## Graphical abstract

Prelamin A interferes with the activation of the Fanconi anemia pathway during DNA replication. On the one hand, prelamin A disrupts the recruitment of FANCD2 to active replication forks under normal conditions, and on the other hand, it impedes the binding of FANCD2 and RAD51 to stalled replication forks under replication stress.

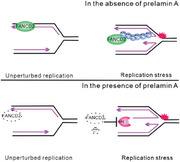

## 1 Introduction

The alternative splicing products of the lamin A/C (*LMNA*) gene, lamin A and lamin C, are major components of the nuclear skeleton.^[^ ^1^ ^]^ Mature lamin A is produced by posttranslational processing of the initial translation product prelamin A.^[^ ^2^ ^]^ Mutations in the *LMNA* gene are closely related to Hutchinson–Gilford progeria syndrome (HGPS). The most widely studied mutation is c.1824C > T (G608G), which licenses an abnormal splice site that abolishes the cleavage site of the zinc metalloproteinase STE24 (ZMPSTE24) from prelamin A, resulting in a truncated and abnormal form lamin A called progerin.^[^ ^3^ ^]^ In addition, defective prelamin A processing caused by ZMPSTE24 deficiency also leads to premature aging.^[^ ^4^ ^]^ Premature senescence associated with lamin A dysfunction is largely attributed to DNA damage accumulation and genomic instability.^[^ ^5^ ^]^

Replication stress, defined as the hindrance of replication progression, is an important source of DNA damage and poses a threat to genome integrity and stability.^[^ ^6^ ^]^ Although A‐type lamins have long been known to be involved in DNA replication, their effect on DNA replication has only gradually been revealed in recent years. Prelamin A and progerin compete with mature lamin A and lamin C for binding to the replication factor proliferating cell nuclear antigen (PCNA), altering the localization of PCNA and leading to its detachment from the replication fork, thereby affecting replication progression.^[^ ^7^ ^]^ Furthermore, DNA fiber assays directly confirmed that progerin increased the number of stalled replication forks and enhanced MRE11 nuclease‐mediated fork degradation.^[^ ^8^ ^]^ While studies have shown that dysfunctional lamin A can interfere with DNA replication and impact the replication stress response, the specific mechanisms by which this occurs and the implications for genome stability remain to be elucidated.

Fanconi anemia (FA) is a genetically heterogeneous bone marrow failure syndrome caused by biallelic mutations in any of the 22 FA genes.^[^ ^9^ ^]^ FA proteins and FA‐related proteins cooperate to repair DNA interstrand crosslinking (ICL); thus, hypersensitivity to ICL inducers is a distinctive feature of FA cells.^[^ ^10^ ^]^ Since breast cancer type 1 susceptibility protein (BRCA1) and breast cancer type 2 susceptibility protein (BRCA2) are also FA proteins, the FA pathway is also called the FA/BRCA pathway.^[^ ^9^ ^]^ The functions of FA/BRCA proteins, however, are not limited to repairing ICLs; these proteins are also important players in the cellular response to a variety of replication barriers.^[^ ^11^ ^]^ Replication fork reversal is a replication stress response mechanism that prevents unrestrained progression across template lesions and facilitates fork rescue and restart by forming a four‐way junction through the backtracking and annealing of nascent strands.^[^ ^12^ ^]^ Despite the beneficial effects of fork reversal, the regressed arms are susceptible to nucleolytic cleavage, which triggers fork collapse.^[^ ^13^ ^]^ RAD51 recombinase binds at the reversed fork to form RAD51 filaments that protect the nascent strand from degradation by MRE11 nuclease and DNA2 nuclease, and BRCA2, BRCA1, FA complementation group D2 (FANCD2), and FA complementation group A (FANCA) protect the replication fork by stabilizing RAD51 filaments.^[^ ^14^ ^]^

FA/BRCA pathway dysfunction results in a range of cellular senescence‐associated phenotypes, including p53‐p21‐mediated G0/G1 arrest induced by constitutively activated DNA damage response signaling, the accumulation of reactive oxygen species, and the senescence‐associated secretory phenotype.^[^ ^15^ ^]^ FA gene downregulation accompanies both replicative senescence and oncogene‐induced senescence.^[^ ^16^ ^]^ Considering the role of the FA/BRCA pathway in maintaining genome stability, especially in preventing replication stress‐induced genomic instability, investigating whether this pathway is involved in lamin A dysfunction‐associated senescence is of interest.

Our study focused on the effect of lamin A dysfunction on the replication stress response and revealed that stalled fork deprotection caused by FA/BRCA pathway inactivation is an important cause of genomic instability, which leads to persistent activation of the p53‐p21 cell cycle arrest axis and cellular senescence.

## 2 Results

### 2.1 Prelamin A Interferes with the Replication Stress Response and Disrupts Genome Stability

**Figure 1:**
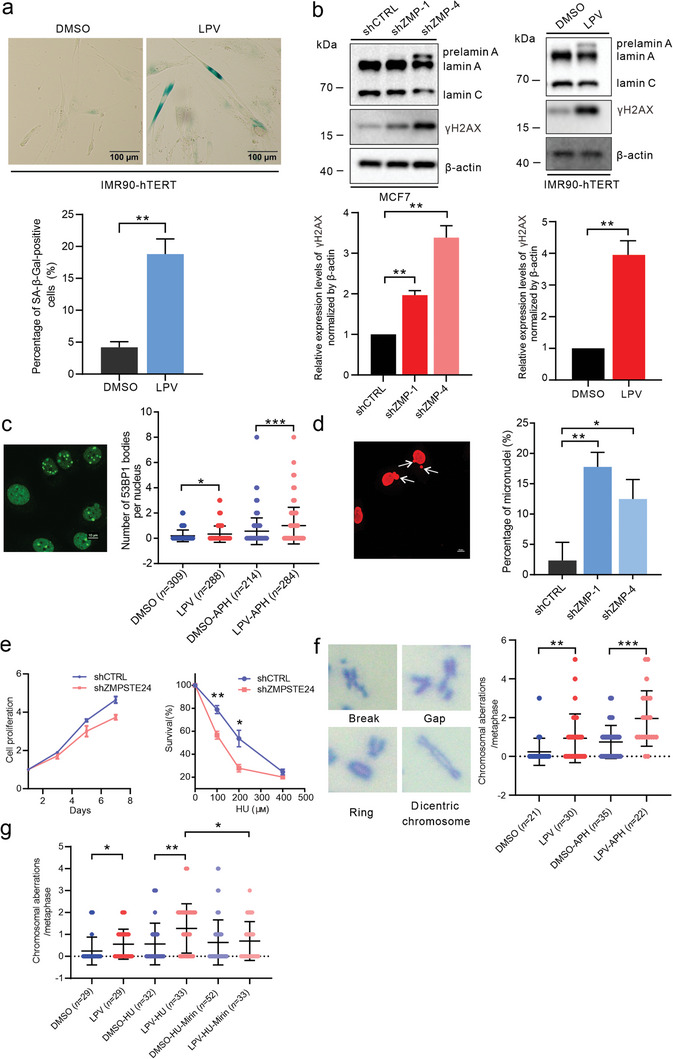
Replication stress response defects and genomic instability in prelamin A‐expressing cells. a) SA‐β‐gal staining of IMR90‐hTERT fibroblasts treated with DMSO or LPV (20 µm) for 2 weeks. Scale bars: 100 µm. The percentage of SA‐β‐gal‐positive cells was calculated. *n* = 3. b) The level of γH2AX in prelamin A‐expressing (*ZMPSTE24*‐knockdown or LPV‐treated) cells was measured by western blotting. *n* = 3. c) Immunofluorescence staining of 53BP1 in control and prelamin A‐expressing (LPV‐treated) cells with or without APH (0.4 µm, 24 h) treatment. Scale bars: 10 µm. The number of 53BP1 bodies per nucleus was calculated. d) Immunofluorescence staining of lamin A/C in shCTRL and shZMPSTE24 cells. Scale bars: 10 µm. The percentage of micronuclei was calculated. *n* = 3. e) The proliferation of shCTRL and shZMPSTE24 cells was measured (left). The viability of shCTRL and shZMPSTE24 cells treated with the indicated concentrations of HU was measured (right). *n* = 3. f) Chromosomal aberrations, including breaks, gaps, rings, and dicentric chromosomes were analyzed for each metaphase in control and prelamin A‐expressing (LPV‐treated) cells with or without APH (0.3 µm, 16 h) treatment. g) Chromosomal aberrations were analyzed for each metaphase in control and prelamin A‐expressing (LPV‐treated) cells with or without HU (4 mм, 4 h) and mirin (50 µm, 4 h) treatment. Quantitative analysis results are shown as the mean ± SD. *P* values were determined by unpaired Student's *t‐*test (a, b, d, and e) and the Mann‒Whitney test (c, f, and g). ^*^ *p* < 0.05; ^**^ *p* < 0.01; ^***^ *p* < 0.001.

Diploid fibroblasts undergo replicative senescence after a limited number of cell divisions in vitro, which is primarily driven by progressive telomere shortening.^[^ ^17^ ^]^ Restoration of telomerase reverse transcriptase (TERT) has been shown to enable diploid fibroblasts to bypass replicative senescence.^[^ ^17^ ^]^ Primary cells cultured in vitro spontaneously and gradually accumulate prelamin A during passage.^[^ ^18^ ^]^ To eliminate the effects of replicative senescence and spontaneous prelamin A accumulation, we generated IMR90 fibroblasts that stably express the human *TERT* gene, hereafter referred to as IMR90‐hTERT fibroblasts (Figure S1a, Supporting Information). HIV protease inhibitors such as lopinavir (LPV) have been shown to inhibit the activity of ZMPSTE24, leading to the abnormal accumulation of prelamin A.^[^ ^19^ ^]^ Senescence‐associated β‐galactosidase (SA‐β‐gal) activity was significantly enhanced in IMR90‐hTERT fibroblasts continuously treated with LPV, demonstrating that prelamin A drives primary cells to enter the senescent state (**Figure** **1**a).

Prelamin A‐expressing MCF7 cells exhibited decreased viability, as indicated by reduced colony formation efficiency (Figure S1b, Supporting Information). We also examined DNA synthesis using the thymidine analog 5‐ethynyl‐2′‐deoxyuridine (EdU), which can be incorporated into nascent DNA and effectively detected via a click reaction.^[^ ^20^ ^]^ The reduced EdU incorporation rate in prelamin A‐expressing cells indicates suppression of cell proliferation (Figure S1c, Supporting Information).

In addition to inducing prelamin A expression using LPV, we generated two *ZMPSTE24*‐knockdown MCF7 cell lines by short hairpin RNA (shRNA) transduction (Figure S1d, Supporting Information). Prelamin A expression led to an increase in the level of phosphorylated histone H2AX (γH2AX) in both IMR90‐hTERT fibroblasts and MCF7 cells, indicating spontaneous replication stress and/or DNA damage (Figure 1b). Replication stress is an important source of DNA damage, and to explore the effect of prelamin A on DNA replication, we examined p53 binding protein 1 (53BP1) body formation. Underreplicated DNA that escapes checkpoint surveillance can be inherited by daughter cells and induces the formation of 53BP1 bodies to prevent the exacerbation of DNA damage; thus, 53BP1 bodies can be used as a marker of replication problems.^[^ ^21^ ^]^ We found that short‐term treatment with LPV caused an increase in the number of 53BP1 bodies, especially after blocking DNA replication at common fragile sites with a low dose of the DNA polymerase inhibitor aphidicolin (APH) (Figure 1c). After the end of mitosis, lagged chromatids or chromosome fragments form micronuclei with membrane structures, and dysregulation of DNA replication and repair factors is closely related to the induction of micronuclei.^[^ ^22^ ^]^ The increased number of micronuclei in *ZMPSTE24*‐knockdown cells is likely also a consequence of replication stress (Figure 1d). Consistent with the attenuation of clonogenicity in LPV‐treated MCF7 cells, ZMPSTE24‐deficient MCF7 cells exhibited proliferation defects in the absence of exogenous replication stress (Figure 1e). Upon induction by the ribonucleotide reductase inhibitor hydroxyurea (HU), the survival of *ZMPSTE24*‐knockdown cells was severely affected (Figure 1e). These results indicate spontaneous accumulation of replication stress and a reduced tolerance to exogenous replication stress in prelamin A‐expressing cells.

We next investigated the effect of prelamin A on the maintenance of genome stability under normal conditions and under conditions of disrupted DNA replication. Prelamin A‐expressing cells showed an increased number of spontaneous chromosome aberrations, mainly breaks and gaps but also rings and dicentric chromosomes (Figure 1f,g). Upon the induction of replication stress by APH or HU treatment, the number of chromosome aberrations per cell was significantly increased in prelamin A‐expressing cells compared with control cells (Figure 1f,g). High or sustained levels of replication stress prompt the resection of unprotected stalled replication forks by the MRE11 nuclease.^[^ ^14b^ ^]^ Progerin interferes with normal replication progression, and inhibition of MRE11 activity can alleviate the replication stress elicited by progerin.^[^ ^8^ ^]^ We hypothesized that the failure of prelamin A‐expressing cells to maintain genome stability under replication stress might be due to the uncontrolled resection of stalled replication forks by MRE11; therefore, we inhibited MRE11 activity while treating cells with HU. As expected, treatment with the MRE11 inhibitor mirin alleviated HU‐induced genomic instability in prelamin A‐expressing cells (Figure 1g). These results confirm that prelamin A interferes with the protective mechanisms of replication forks under replication stress and therefore disrupts genome stability.

### 2.2 The Abundance of FA/BRCA Proteins is Reduced in Prelamin A‐Expressing Cells

**Figure 2:**
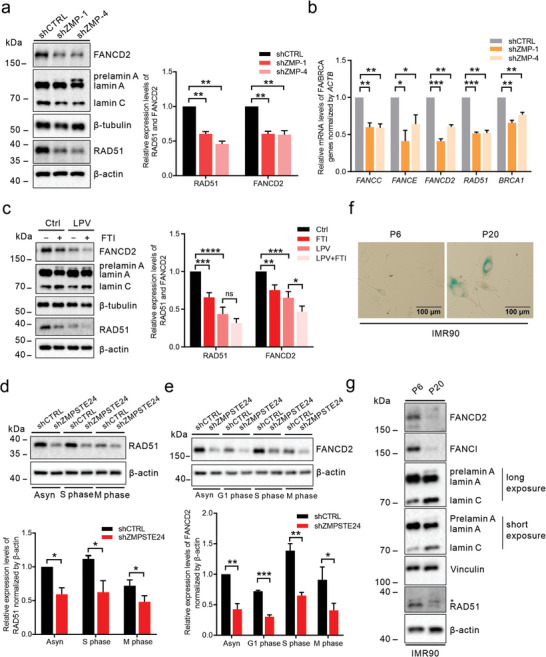
Downregulation of the FA/BRCA gene network in prelamin A‐expressing cells. a) The expression of FANCD2 and RAD51 in *ZMPSTE24*‐knockdown cells was measured by western blotting. *n* = 3. b) mRNA levels of FA/BRCA genes in *ZMPSTE24*‐knockdown cells were measured by qPCR. *n* = 3. c) Western blotting was used to measure the expression of FANCD2 and RAD51 in cells treated with FTI (3 µm, 48 h), LPV (20 µm, 6 days) or LPV + FTI. The farnesylated prelamin A generated by LPV treatment (lane 3) migrated more rapidly than the nonfarnesylated prelamin A generated by FTI treatment (lane 2 and lane 4). *n* = 3. d) The expression of RAD51 in asynchronous, S‐phase‐synchronized and M‐phase‐synchronized control and *ZMPSTE24*‐knockdown cells was measured by western blotting. Asyn, asynchronous. *n* = 3. e) The expression of FANCD2 in asynchronous, G1‐phase‐synchronized, S‐phase‐synchronized and M‐phase‐synchronized control and *ZMPSTE24*‐knockdown cells was measured by western blotting. Asyn, asynchronous. *n* = 3. f) SA‐β‐gal staining of IMR90 fibroblasts at early (P6) and late (P20) passages. Scale bar: 100 µm. g) The protein levels of prelamin A, FANCD2, FANCI and RAD51 in IMR90 fibroblasts at early (P6) and late (P20) passages were detected by western blotting. Quantitative analysis results are shown as the mean ± SD. *P* values were determined by unpaired Student's *t‐*test (a, b, d, and e) and one‐way ANOVA (c). ns, not significant; ^*^ *p* < 0.05; ^**^ *p* < 0.01; ^***^ *p* < 0.001; ^****^ *p* < 0.0001.

We proceeded to explore the mechanism by which prelamin A leads to the accumulation of endogenous replication stress and hypersensitivity to replication stress inducers. A functional FA/BRCA pathway is required for DNA replication progression under normal conditions and for the stabilization and restart of stalled replication forks under replication stress.^[^ ^11^ ^]^ We wondered whether the FA/BRCA pathway, which is closely related to the maintenance of genome integrity, is regulated by prelamin A. The protein and transcriptional levels of the FA/BRCA pathway members FANCD2 and RAD51 were significantly decreased in *ZMPSTE24*‐knockdown cells (**Figure** **2**a,b). In addition, the transcript levels of FA complementation group C (FANCC) and FA complementation group E (FANCE), components of the FA core complex, and BRCA1, a homologous recombination pathway repair gene, were also significantly downregulated by prelamin A expression (Figure 2b). Inhibition of ZMPSTE24 activity by LPV treatment in IMR90‐hTERT fibroblasts also diminished the expression of the FA/BRCA pathway components FANCD2, RAD51, FANCC, FA complementation group I (FANCI), and BRCA1 (Figure S2a,b, Supporting Information).

Similar to the truncated lamin A protein progerin, prelamin A produced by ZMPSTE24 depletion or inhibition retains a farnesyl lipid modification on the last cysteine residue.^[^ ^2^ ^]^ Blocking farnesylation has been shown to prevent and/or ameliorate detrimental phenotypes in HGPS cells, HGPS mouse models, and HGPS patients.^[^ ^3^, ^23^ ^]^ Since the first step in the posttranslational processing of prelamin A, farnesylation of the *CaaX* motif, is a prerequisite for subsequent enzymatic modifications, treatment with farnesyltransferase inhibitors (FTIs) induces the production of nonfarnesylated prelamin A.^[^ ^19^ ^]^ We subjected cells to FTI‐277 treatment to investigate the requirement of farnesylation for the regulation of FANCD2 and RAD51 levels by prelamin A. Nonfarnesylated prelamin A also resulted in a decrease in the RAD51 and FANCD2 protein levels, and inhibition of farnesylation in the background of LPV treatment did not reverse the downregulation of RAD51 and FANCD2 (Figure 2c). These results indicate that farnesylation is dispensable for the regulation of the FA/BRCA pathway by prelamin A.

The levels of the FA core complex components (except FANCC and FANCF), as well as those of FANCD2, FANCI, BRCA1, and RAD51, are cell cycle‐related, with most peaking during the S phase.^[^ ^24^ ^]^ Therefore, we examined the RAD51 and FANCD2 levels in synchronized cells and showed that silencing the *ZMPSTE24* gene caused a decrease in RAD51 and FANCD2 expression throughout the cell cycle (Figure 2d,e). This finding indicates that alterations in the cell cycle may contribute to but are not the only cause of the downregulation of the FA/BRCA pathway in prelamin A‐expressing cells.

The detection of prelamin A in tissues and cells obtained from aged individuals suggests a potential association between defective prelamin A processing and physiological aging.^[^ ^18a^ ^]^ The accumulation of prelamin A has also been observed during cellular replicative senescence, an in vitro model of physiological aging.^[^ ^18a^ ^]^ Subsequently, we explored the correlation between prelamin A accumulation and the downregulation of the FA/BRCA pathway during replicative senescence by utilizing primary IMR90 fibroblasts at both early and late passages. At the late passage, IMR90 fibroblasts exhibited a flattened morphology and increased staining for SA‐β‐gal (Figure 2f). Prelamin A was almost undetectable in IMR90 fibroblasts at passage 6 but accumulated at passage 20, and there was a significant decrease in the levels of FANCD2, FANCI, and RAD51, which underscores a concurrent process in which prelamin A accumulation and FA/BRCA pathway downregulation occur during replicative senescence (Figure 2g).

Could dysregulation of the FA/BRCA pathway in turn affect prelamin A processing? To address this question, we examined the expression of prelamin A in FANCD2‐deficient FA patient cell line PD20 and PD20 cells supplemented with wild‐type FANCD2 (PD20+D2). The results reveal that the absence of FANCD2, a critical component of the FA pathway, did not lead to aberrant production of prelamin A (Figure S2c, Supporting Information).

### 2.3 Prelamin A Inhibits the Activation of the FA/BRCA Pathway During Both Unperturbed Replication and Replication Stress Conditions

**Figure 3:**
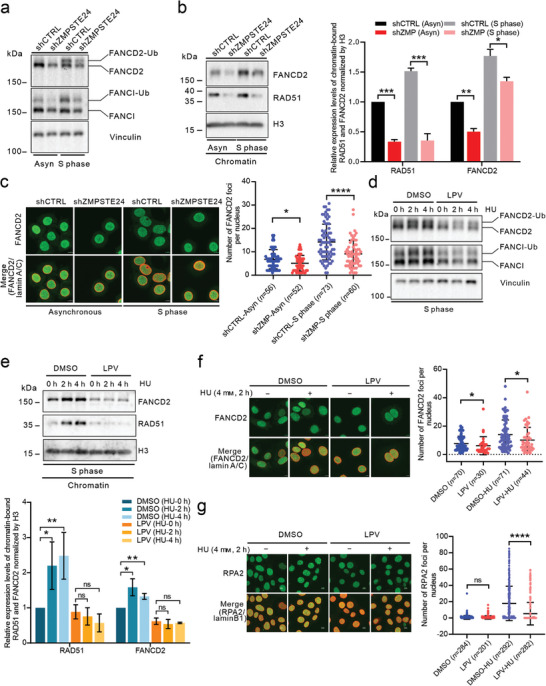
Attenuated activation of the FA/BRCA pathway under normal conditions and upon replication stress in prelamin A‐expressing cells. a) The monoubiquitination levels of FANCD2 and FANCI in asynchronous and S‐phase‐synchronized control and *ZMPSTE24*‐knockdown cells were measured by western blotting. b) The expression of FANCD2 and RAD51 in the chromatin‐bound protein fraction of asynchronous and S‐phase‐synchronized control and *ZMPSTE24*‐knockdown cells was measured by western blotting. *n* = 3. c) FANCD2 foci formation in asynchronous and S‐phase‐synchronized control and *ZMPSTE24*‐knockdown cells. Only the nuclei containing FANCD2 foci were counted. Scale bars: 10 µm. d) Monoubiquitination levels of FANCD2 and FANCI in S‐phase‐synchronized control (DMSO) and prelamin A‐expressing (LPV, 20 µm, 6 days) cells treated with HU (4 mм). e) The expression of FANCD2 and RAD51 in the chromatin‐bound protein fraction of S‐phase‐synchronized control (DMSO) and prelamin A‐expressing (LPV, 20 µm, 6 days) cells treated with HU (4 mм). *n* = 4 or 5. f) FANCD2 foci formation in control (DMSO) and prelamin A‐expressing (LPV, 20 µm, 6 days) cells with or without HU (4 mм, 2 h) exposure. Only the nuclei containing FANCD2 foci were counted. Scale bars: 10 µm. g) RPA2 foci formation in S‐phase‐synchronized control (DMSO) and prelamin A‐expressing (LPV, 20 µm, 6 days) cells with or without HU (4 mм, 2 h) exposure. Scale bars: 10 µm. Quantitative analysis results are shown as the mean ± SD. *P* values were determined by unpaired Student's *t‐*test (b and e) and the Mann‒Whitney test (c, f, and g). ns, not significant; ^*^ *p* < 0.05; ^**^ *p* < 0.01; ^***^ *p* < 0.001; ^****^ *p* < 0.0001.

In the absence of external replication stress, FANCD2 undergoes monoubiquitination during the S phase of the cell cycle and forms nuclear foci that are colocalized with BRCA1 and RAD51.^[^ ^25^ ^]^ FANCI, like its partner FANCD2, is also enriched in active replication forks.^[^ ^26^ ^]^ Based on these studies, we first investigated the effect of prelamin A on FA/BRCA pathway activation under unperturbed S phase conditions. In control cells, the levels of mono‐ubiquitinated FANCD2 and FANCI were markedly elevated during the S phase (**Figure** **3**a). However, in ZMPSTE24‐depleted cells, although the levels of mono‐ubiquitinated FANCD2 and FANCI also increased during the S phase, they were notably lower than those in control cells (Figure 3a). We further isolated the chromatin‐bound protein fraction to investigate the enrichment of FANCD2 and RAD51 on chromatin. The purity of the extracted chromatin‐bound protein fractions is shown in Figure S3 (Supporting Information). Knockdown of *ZMPSTE24* significantly reduced RAD51 and FANCD2 binding to chromatin in both asynchronous and S‐phase‐synchronized cells (Figure 3b). The percentage of FANCD2 foci‐positive cells was similar between control and *ZMPSTE24*‐knockdown cells, with ≈40% in asynchronous cells and 70% in S‐phase‐synchronized cells. However, the number of FANCD2 foci formed in *ZMPSTE24*‐knockdown cells was significantly lower than that in control cells, particularly in synchronized S phase cells (Figure 3c).

We next focused on the response of FA/BRCA proteins to perturbed replication in the presence of prelamin A. BRCA1, BRCA2, the mono‐ubiquitinated FANCD2‐FANCI complex, and FANCA collectively play crucial roles in stabilizing RAD51 filaments to prevent excessive nuclease processing of stressed replication forks.^[^ ^14a–c^ ^]^ The monoubiquitination levels of both FANCD2 and FANCI were significantly increased in control cells following HU induction, indicating an active response to replication stress (Figure 3d). However, in the presence of prelamin A, this response was notably altered, with the monoubiquitination levels of FANCD2 and FANCI remaining largely unchanged after HU induction (Figure 3d). In the isolated S‐phase chromatin‐bound protein fraction, HU induction led to a significant increase in the levels of chromatin‐bound FANCD2 and RAD51 in control cells (Figure 3e). However, in cells expressing prelamin A, the recruitment of FANCD2 and RAD51 to chromatin in response to replication stress was almost completely inhibited (Figure 3e). Consistently, the presence of prelamin A was found to impact FANCD2 foci formation upon HU induction (Figure 3f).

The inhibition of FANCD2 and FANCI monoubiquitination by prelamin A in the S phase and under replication stress was indeed associated with decreased levels of FANCD2 and FANCI, as well as reduced levels of components of the FA core complex responsible for the catalytic monoubiquitination of FANCD2 and FANCI. Additionally, there could be other factors contributing to the inadequate activation of the FA/BRCA pathway in cells expressing prelamin A. When replication is perturbed, lamin A/C binds to newly synthesized DNA and recruits the replication protein A (RPA) complex, FANCD2, and RAD51.^[^ ^27^ ^]^ RPA serves as the first responder and facilitates the chromatin loading of BRCA1/BRCA2, FANCD2, and RAD51 upon replication stress.^[^ ^28^ ^]^ The expression of prelamin A may interfere with this important function of lamin A/C. Consistent with our hypothesis, the number of RPA foci formed after HU exposure was significantly lower in S‐phase‐synchronized prelamin A‐expressing cells than in control cells (Figure 3g). These results show that the FA/BRCA pathway is not efficiently activated in prelamin A‐expressing cells when replication stress occurs.

### 2.4 FA/BRCA Genes are Downregulated by Prelamin A in an RB/E2F‐Dependent Manner

The disruption of the nuclear lamina results in reduced global transcription through several mechanisms, such as the sequestration of certain transcription factors, decreased activity of RNA polymerase II, modulation of miRNA expression, and alterations in chromatin organization and modification.^[^ ^29^ ^]^ Prelamin A has been reported to sequester the transcription factor sterol regulatory element‐binding protein 1 (SREBP1) in the nuclear lamina, thereby inhibiting the transcription of SREBP1 target genes.^[^ ^30^ ^]^ Prelamin A also leads to an increase in the heterochromatin mark H3K9me3 and a decrease in H4K16ac associated with transcriptional activation.^[^ ^31^ ^]^ Therefore, the transcriptional repression of FA/BRCA genes by prelamin A is not specific. We then aimed to investigate the underlying causes of the decreased transcription of FA/BRCA genes induced by prelamin A among the numerous mechanisms through which prelamin A represses transcription. The currently known regulation of FA/BRCA genes at the transcriptional level involves the retinoblastoma (RB)/E2F pathway.^[^ ^16^, ^32^ ^]^ E2F transcription factor 1 (E2F1) binds to the E2F elements in the FA gene promoter to activate transcription, and the activated hypophosphorylated RB forms a complex with E2F1 to block the binding of E2F1 to the promoter and repress E2F1‐mediated transcription.^[^ ^16a^ ^]^ E2F transcription factor 4 (E2F4) can form the transcriptionally repressed DREAM (DP, RB‐like, E2F, and MuvB) complex with the RB‐related proteins p107 or p130 and the MuvB core complex.^[^ ^33^ ^]^ In this complex, E2F4 binds to the CDE/CHR motif on the promoter of target genes, including most FA/BRCA genes, to repress their expression.^[^ ^32^ ^]^

**Figure 4:**
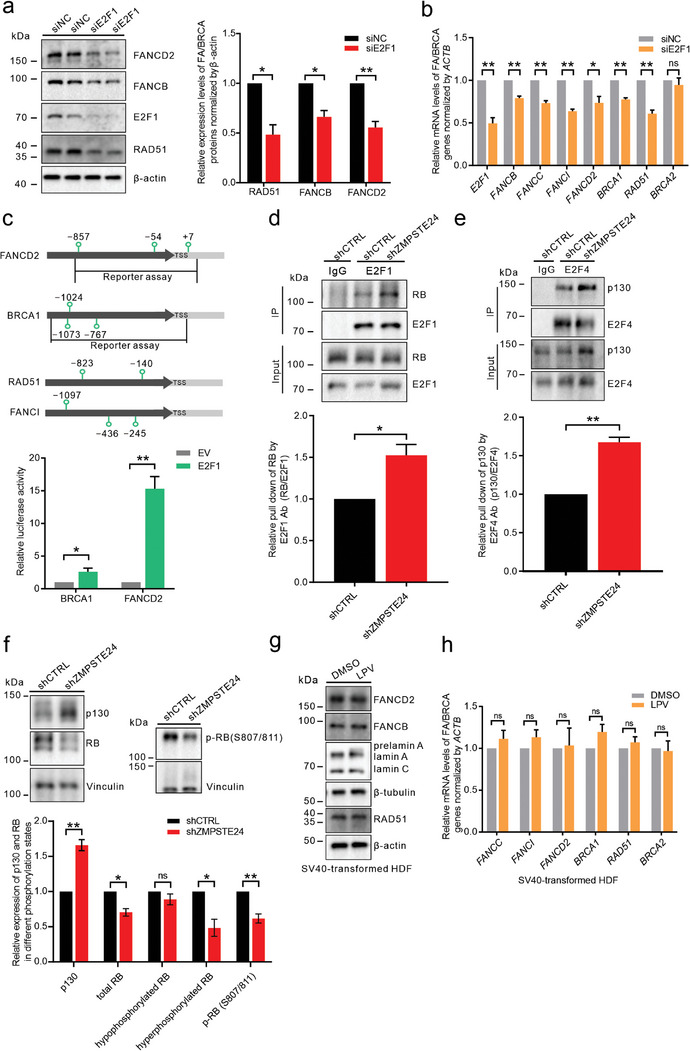
The repressive RB/E2F complex mediates the downregulation of the FA/BRCA pathway by prelamin A. a) The expression of FANCD2, FANCB, and RAD51 in *E2F1*‐knockdown cells was measured by western blotting. *n* = 3. b) mRNA levels of FA/BRCA genes in *E2F1*‐knockdown cells were measured by qPCR. *n* = 3. c) The three most likely binding sites of E2F transcription factors predicted by the JASPAR database (https://jaspar.genereg.net/) in the FA/BRCA gene promoter as well as in the downstream region proximal to the transcription start site (TSS) are annotated in the upper panel. In the dual luciferase reporter assay, the E2F1 expression vector, firefly luciferase reporter vector containing the FA/BRCA gene promoter, and *Renilla* luciferase reporter vector were cotransfected into cells in the experimental group, and the E2F1 expression vector was replaced with the empty vector in cells in the control group. The ratio of the normalized luciferase activity in the experimental and control groups was defined as the fold change in expression. *n* = 3. d) Whole‐cell extracts were subjected to immunoprecipitation with an anti‐E2F1 antibody prior to immunoblotting to evaluate E2F1 and RB expression in the input and IP samples. *n* = 3. e) Whole‐cell extracts were subjected to immunoprecipitation with an anti‐E2F4 antibody prior to immunoblotting to evaluate E2F4 and p130 expression in the input and IP samples. *n* = 3. f) The expression of p130, RB, and p‐RB (S807/811) in control and *ZMPSTE24*‐knockdown cells was measured by western blotting. The larger band of RB represents hyperphosphorylated RB, and the smaller band of RB represents hypophosphorylated RB. *n* = 3. g) The expression of FANCD2, FANCB, and RAD51 in DMSO‐ and LPV (20 µm, 6 days)‐treated SV40‐transformed HDFs was measured by western blotting. h) mRNA levels of FA/BRCA genes in LPV (20 µm, 6 days)‐treated SV40‐transformed HDFs were measured by qPCR. *n* = 3. Quantitative analysis results are shown as the mean ± SD. *P* values were determined by unpaired Student's *t‐*test. ns, not significant, ^*^ *p* < 0.05; ^**^ *p* < 0.01.

In previous studies, the suppressive effect of E2F4 on most FA/BRCA genes has been established.^[^ ^32^ ^]^ However, the promoting effect of E2F1 on FA/BRCA genes has only been validated in a limited number of genes.^[^ ^16a^ ^]^ Therefore, we aimed to provide a comprehensive analysis of the regulatory role of E2F1 in a broader spectrum of genes within the FA/BRCA pathway. siRNA‐mediated silencing of the *E2F1* gene resulted in the downregulation of both the protein and transcription levels of multiple FA/BRCA genes (**Figure** **4**a,b). Using the JASPAR database, we characterized the binding profiles of E2F transcription factors at the promoters of FA/BRCA genes (Figure 4c). Furthermore, we confirmed the E2F1‐mediated activation of the FANCD2 and BRCA1 promoters by dual‐luciferase reporter assays (Figure 4c).

To investigate the potential involvement of RB/E2F in the downregulation of FA/BRCA gene expression mediated by prelamin A, we conducted immunoprecipitation assays to examine the formation of endogenous RB‐E2F1 and p130‐E2F4 complexes. Interestingly, our results show that prelamin A expression led to increased formation of both the RB‐E2F1 and p130‐E2F4 complexes (Figure 4d,e). Cyclin dependent kinase 1 (*CDK1*) and cyclin A2 (*CCNA2*) are known to be directly regulated by the RB/E2F complex.^[^ ^33^ ^]^ In prelamin A‐expressing cells, the decreased transcript levels of *CDK1* and *CCNA2* support the conclusion that the formation of the RB/E2F repressive complex is promoted by prelamin A (Figure S4, Supporting Information). Prelamin A expression led to a significant increase in the p130 protein level, and this increase may contribute to the enhanced interaction between E2F4 and p130 (Figure 4f). In contrast, the RB protein level was decreased in prelamin A‐expressing cells (Figure 4f). Notably, western blot analysis revealed that hypophosphorylated RB (faster migrating) remained unchanged in the presence of prelamin A, but the amount of hyperphosphorylated RB (slower migrating) was greatly reduced by prelamin A (Figure 4f). We further confirmed the downregulation of the hyperphosphorylated form of RB by prelamin A using a phosphorylation‐specific antibody (Figure 4f).

Simian virus 40 (SV40) promotes cell transformation through its encoded large T antigen and small T antigen.^[^ ^34^ ^]^ The LxCxE motif of the large T antigen binds to RB family members (RB, p107, and p130) to dissociate them from E2F proteins.^[^ ^34^ ^]^ We next induced prelamin A in SV40‐immortalized human dermal fibroblasts (HDFs) and found that the protein and transcript levels of FA/BRCA pathway genes were not affected (Figure 4g,h). This observation suggests that the regulation of the FA/BRCA gene network by prelamin A is mediated through the involvement of RB family members.

### 2.5 FA/BRCA Pathway Downregulation is an Upstream Event of p53‐p21 Axis Activation During Prelamin A Induction

**Figure 5:**
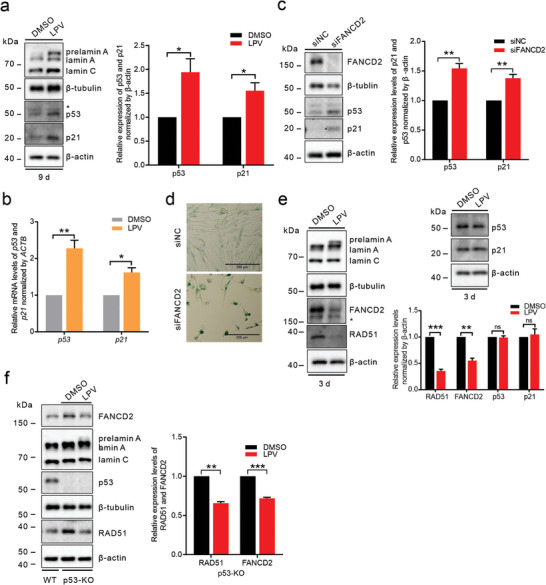
Downregulation of the FA/BRCA pathway occurs earlier than activation of the p53‐p21 axis during prelamin A induction. a) The expression of p21 and p53 in DMSO‐ and LPV (20 µm, 9 days)‐treated IMR90‐hTERT fibroblasts was measured by western blotting. *n* = 3. b) mRNA levels of p21 and p53 in DMSO‐ and LPV (20 µm, 9 days)‐treated IMR90‐hTERT fibroblasts were measured by qPCR. *n* = 3. c) The expression of p21 and p53 in *FANCD2*‐knockdown IMR90‐hTERT fibroblasts was measured by western blotting. *n* = 3. d) SA‐β‐gal staining of control and *FANCD2*‐knockdown IMR90‐hTERT fibroblasts. Scale bars: 200 µm. e) The protein levels of FANCD2, RAD51, p21, and p53 in DMSO‐ and LPV (20 µm, 3 days)‐treated IMR90‐hTERT fibroblasts were measured by western blotting. *n* = 3. f) The expression of FANCD2 and RAD51 in DMSO‐ and LPV (20 µm, 6 days)‐treated p53‐knockout IMR90‐hTERT fibroblasts was measured by western blotting. *n* = 3. Quantitative analysis results are shown as the mean ± SD. *P* values were determined by unpaired Student's *t‐*test. ns, not significant, ^*^ *p* < 0.05; ^**^ *p* < 0.01, ^***^p < 0.001.

The activation of the p53‐p21 axis represents a fundamental cellular response to DNA damage, which triggers a temporary pause in the cell cycle, enabling the repair of DNA damage.^[^ ^35^ ^]^ However, sustained or excessive activation of the p53‐p21 axis due to persistent DNA damage can drive cells into a state of irreversible growth arrest, referred to as cell senescence.^[^ ^35^ ^]^ p53 signaling is hyperactivated in *Zmpste24* knockout mice, and downstream p53 target genes, including p21, are significantly upregulated, although the expression level of p53 itself is not affected.^[^ ^36^ ^]^ LPV treatment of IMR90‐hTERT fibroblasts also resulted in activation of the p53‐p21 axis, as both p53 and p21 were upregulated at the protein and transcriptional levels (**Figure** **5**a,b).

Activation of the p53‐p21 axis has been reported to downregulate the FA/BRCA gene network by facilitating the formation of RB‐E2F repressive complexes.^[^ ^32^, ^37^ ^]^ The inhibitory effect of p53 on the FA/BRCA gene network was validated through the observation of decreased levels of FA/BRCA genes in cells exhibiting p53 accumulation following treatment with the Mdm2 inhibitor Nutlin‐3A (Figure S5a,b, Supporting Information). Conversely, elevated levels of FANCD2 and RAD51 were noted in p53‐knockout cells (Figure S5c, Supporting Information). However, we cannot conclude based on these results that prelamin A facilitates the formation of the RB‐E2F complex by activating the p53‐p21 axis, thereby suppressing the FA/BRCA pathway. This is because the persistent replication stress and accumulation of DNA damage in FA and FA‐like cells due to FA/BRCA pathway defects sequentially activate the p53/p21 axis.^[^ ^15^, ^38^ ^]^ Consistent with this observation, we detected increased levels of p53 and p21 and increased SA‐β‐gal activity in *FANCD2*‐knockdown IMR90‐hTERT fibroblasts (Figure 5c,d). Previous studies,^[^ ^15^, ^32^, ^38^ ^]^ along with the results obtained in our research, have provided evidence supporting a positive feedback loop between the activation of the p53‐p21 axis and the inhibition of the FA/BRCA pathway.

To investigate the sequence of p53/p21 axis activation and FA/BRCA pathway repression in response to prelamin A induction, we treated IMR90‐hTERT fibroblasts with a shorter duration of LPV. Our findings reveal a significant downregulation of FANCD2 and RAD51 expression at this specific time point, while no notable changes were observed in the expression levels of p53 and p21 (Figure 5e). Moreover, the induction of prelamin A in the absence of p53 also led to the downregulation of FANCD2 and RAD51 (Figure 5f). These data indicate that prelamin A initially inhibits FA/BRCA gene expression in a p53‐independent manner and that subsequent p53‐p21 activation perpetuates the repression of FA/BRCA genes. This sequence of events implies that functional impairment of the FA/BRCA pathway is an important link in prelamin A‐driven cellular senescence.

## 3 Conclusion and Discussion

**Figure 6:**
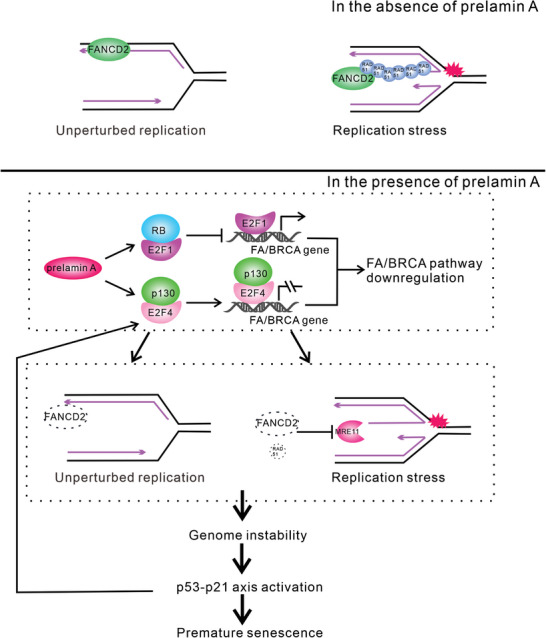
Schematic model of prelamin A‐induced premature senescence. Downregulation of the FA/BRCA pathway mediated by RB‐E2F inhibitory complexes leads to failure of effective activation of the replication fork protection mechanism involving the FA/BRCA gene network in response to replication stress, and the deprotected replication forks are subjected to hyper resection by the MRE11 nuclease. Prelamin A also inhibits the recruitment of FANCD2 to unchallenged replication forks, potentially contributing to endogenous replication stress. Dysregulation of the replication stress response leads to genomic instability, excessive activation of the p53‐p21 axis, and permanent cell cycle arrest. The activated p53‐p21 axis further inhibits FA/BRCA gene expression, establishing a positive feedback loop that propels cells expressing prelamin A toward irreversible senescence.

Our study focused on the impact of replication stress on genomic stability in cells expressing prelamin A, highlighting the significance of prelamin A‐mediated suppression of the FA/BRCA pathway in generating endogenous replication stress and promoting the instability of stalled replication forks (**Figure** **6**). Prelamin A transcriptionally suppresses multiple FA/BRCA genes by promoting the formation of RB‐E2F1 and p130‐E2F4 complexes. During normal replication, prelamin A hinders the recruitment of FANCD2 to active replication forks, potentially disrupting the normal progression of replication. Under exogenous replication stress, prelamin A impedes the recruitment of protective factors such as FANCD2, FANCI, and RAD51 to stalled replication forks, facilitating excessive degradation of nascent strands by the MRE11 nuclease. The accumulation of DNA damage during the replication process triggers excessive activation of the p53‐p21 axis, which leads to irreversible cell cycle arrest.

In the absence of exogenous genotoxic stimulation, the increased levels of γH2AX in prelamin A‐expressing cells, as well as the increased formation of 53BP1 bodies and micronuclei, indicate spontaneous replication stress (Figure 1b–d). During unperturbed replication, lamin A binds to replication polymerase, components of the minichromosome maintenance helicase, and PCNA and is a component of the active replisome.^[^ ^39^ ^]^ Disruption of the lamin A‐PCNA interaction by progerin and prelamin A has been shown to result in replication fork stalling.^[^ ^7^ ^]^ In this study, we found that the interference of prelamin A on normal replication may also involve the FA/BRCA pathway. In the absence of external replication stress, prelamin A significantly suppressed the expression of FANCD2 and RAD51 in the S phase, as well as impeding the recruitment of FANCD2, RAD51, and FANCI to chromatin during this phase (Figures 2d,e and 3a–c). During the S phase, monoubiquitinated FANCD2 binds to the replication origin and minichromosome maintenance complex component 3 to ensure the initiation of replication origin.^[^ ^40^ ^]^ The FA/BRCA pathway can also restrict the accumulation of R‐loops caused by collisions between transcription and replication, thereby preventing the escalation of spontaneous replication stress.^[^ ^41^ ^]^ Dysregulation of the FA/BRCA pathway in cells expressing prelamin A is likely to affect the mechanisms involved in maintaining normal replication, but further research is needed to confirm this.

Both the hypersensitivity to replication stress inducers and the large increase in chromosome aberrations upon replication stalling provide evidence that prelamin A exacerbates the detrimental impact of replication stress on genome stability and cell survival (Figure 1e–g). The administration of an MRE11 nuclease inhibitor mitigated HU‐induced genomic instability in prelamin A‐expressing cells (Figure 1g), prompting further investigation into the impact of prelamin A on the protective mechanism of stalled replication forks during replication stress. The protective effects of monoubiquitinated FANCD2 and FANCI, as well as the FA core complex, on stalled replication forks rely on the formation and stabilization of RAD51 nuclear filaments.^[^ ^14c^ ^]^ Prelamin A not only attenuated the expression of FANCD2, FANCI, and RAD51 but also significantly impeded the recruitment of these protective factors to stressed forks (Figures 2 and 3d–f).

The cellular pathways altered during lamin A dysfunction‐associated senescence are not independent, and there are many positive feedback interactions between them. It is difficult to distinguish which alterations are crucial in driving senescence and which are secondary effects of long‐term lamin A dysfunction in patient‐derived cells and genetically engineered cells that intrinsically show obvious senescence phenotypes. The repression of the FA/BRCA network and activation of the p53‐p21 axis are mutual causes.^[^ ^15^, ^32^, ^38^ ^]^ In this study, we induced prelamin A production by LPV treatment, examined the changes in the FA/BRCA pathway and p53‐p21 axis at different time points after induction, and concluded that downregulation of the FA/BRCA pathway occurred earlier than activation of the p53‐p21 axis (Figure 5e). A previous study,^[^ ^32^ ^]^ as well as our results (Figure S5, Supporting Information), confirmed that activation of the p53‐p21 axis further exacerbates the suppression of the FA/BRCA pathway, and this positive feedback irreversibly drives prelamin A‐expressing cells toward senescence.

The discovery of a compromised FA/BRCA pathway in prelamin A‐expressing cells in this study is of significant importance for uncovering the molecular mechanisms that underlie lamin A‐related premature aging diseases. Dysregulation of the FA/BRCA pathway not only results in disruption of normal replication, deprotection of stalled replication forks, and DNA repair defects but also leads to telomere attrition and mitochondrial alterations, which are typical features of senescent cells, including those with lamin‐associated premature senescence.^[^ ^42^ ^]^ Thus, it is plausible that the FA/BRCA pathway could counteract prelamin A‐induced senescence through several mechanisms. Further research into the pathogenesis of FA and the functions of the FA/BRCA pathway may provide valuable insights into the mechanisms of laminopathy induced by lamin A dysfunction.

## 4 Experimental Section

### Cell Lines and Constructs

MCF7 cells, HEK293‐T cells, and SV40‐transformed HDFs were cultured in Dulbecco's modified Eagle's medium (high‐glucose) supplemented with 10% fetal bovine serum (FBS) and 1% penicillin and streptomycin (p/s). IMR90 fibroblasts were maintained in minimal essential medium supplemented with 10% FBS and 1% p/s. PD20 and PD20+D2 cells were cultured in Roswell Park Memorial Institute 1640 medium supplemented with 10% FBS and 1% p/s.

IMR90 fibroblasts were immortalized via the integration of the *hTERT* gene into the genome via lentivirus infection. The *hTERT* gene was cloned and inserted into pCDH‐CMV‐MCS‐EF1‐Neo, and the resulting plasmid was cotransfected with pMD2.G and psPAX2 into HEK293‐T cells using Lipofectamine 2000 (Invitrogen). Both the cells and medium were collected for centrifugation and filtration 48–72 h later. The supernatant was then mixed with medium to infect IMR90 fibroblasts in the presence of polybrene (8 µg mL^−1^), after which the cells were cultured in medium containing G418 (400 µg mL^−1^) for 1–2 weeks. The primers utilized for confirming the successful construction of IMR90‐hTERT fibroblasts are detailed in Table S1 (Supporting Information).

To generate knockdown cell lines, shRNA sequences targeting *ZMPSTE24* were cloned and inserted into the pLKO.1‐puro vector. p53‐knockout cells were generated by inserting the gRNA sequence into lentiCRISPR v2 and transducing cells with the resulting plasmid as described above. The cells were then treated with puromycin (1 µg mL^−1^). The shRNA and gRNA targeting sequences are listed in Table S2 (Supporting Information).

### siRNA Transfection

siRNA transfection was conducted using a ribo*FECT* CP Transfection Kit (RiboBio, C10511‐05). The cells were incubated for 72 h with siRNAs (50 nм) before western blotting or qPCR analysis. The siRNA sequences used are listed in Table S2 (Supporting Information).

### Antibodies and Reagents

Anti‐lamin A/C (Proteintech, 10298‐1‐AP), anti‐lamin A/C (Santa Cruz, sc‐376248), anti‐lamin B1 (Proteintech, 12987‐1‐AP), anti‐β‐actin (ABclonal, AC026), anti‐β‐tubulin (ABclonal, AC021), anti‐vinculin (Proteintech, 26520‐1‐AP), anti‐H2AX‐pS139 (Santa Cruz, sc‐517348), anti‐p53 (Proteintech, 60283‐2‐Ig), anti‐p21 (Proteintech, 10355‐1‐AP), anti‐H3 (Proteintech, 17168‐1‐AP), anti‐E2F1 (Proteintech, 66515‐1‐Ig), anti‐E2F1 (ABclonal, A19579), anti‐E2F4 (Proteintech, 10923‐1‐AP), anti‐RB (ABclonal, A3618), anti‐p130 (ABclonal, A13649), anti‐p‐RB (S807/811) (Proteintech, 30376‐1‐AP), anti‐FANCD2 (Proteintech, 28619‐1‐AP), anti‐FANCD2 (Novus biologicals, NB100‐182), anti‐FANCB (ZEN BIO, 384 281), anti‐FANCI (Proteintech, 67304‐1‐Ig), anti‐RAD51 (Proteintech, 14961‐1‐AP), anti‐53BP1 (ABclonal, A5757), anti‐RPA2 (Santa Cruz, sc‐56770), rabbit control IgG (ABclonal, AC005) and mouse control IgG (ABclonal, AC011) were used.

LPV (MCE, HY‐14588), FTI‐277 (MCE, HY‐15872A), HU (Sigma–Aldrich, H8627), APH (Glpbio, GC10867), colcemid (Glpbio, GC40664), Nutlin‐3A (MCE, HY‐10029), and mirin (MCE, HY‐117693) were used.

### Western Blotting and Immunofluorescence Staining

Western blotting and immunofluorescence staining were performed as described previously.^[^ ^43^ ^]^

### RNA Extraction and qPCR

RNA extraction and qPCR were performed as described previously.^[^ ^43^ ^]^ The qPCR primers used are listed in Table S3 (Supporting Information).

### EdU Incorporation Assay

DNA synthesis was evaluated by an EdU cell proliferation assay kit (Beyotime, C0071). In brief, cells grown on confocal Petri dishes were labeled with EdU (10 µm) for 4 h. After fixation and permeabilization, freshly prepared click reaction solution was added prior to incubation at RT in the dark for 30 min. After washing with PBS, EdU incorporation was observed under a fluorescence microscope.

### Senescence‐Associated β‐galactosidase Staining

Senescence‐associated β‐galactosidase staining was performed according to the manufacturer's instructions (Beyotime, C0602). The cells were fixed for 15 min at RT, washed with PBS, incubated overnight with freshly prepared staining solution at 37 °C, and then examined under a light microscope.

### Cell Viability Assay

The viability of cells after drug treatment was measured according to the instructions of the CellTiter‐Lumi Luminescent Cell Viability Assay Kit (Beyotime, C0065). A total of 1000 MCF7 cells per well were cultured in 96‐well plates, and 24 h later, the cells were treated with different concentrations of HU (0 µm, 100 µm, 200 µm, or 400 µm). Six days later, a detection reagent (100 µL) was added to each well, and the plates were incubated for 10 min at RT. The chemiluminescence intensity was then measured by a Molecular Devices SpectraMax i3x Multi‐Mode Microplate Detection System.

To examine colony formation ability, a total of 1000 MCF7 cells per well were seeded in each well of a 12‐well plate and cultured with medium containing DMSO or LPV (20 µm). Two weeks later, the cells were fixed with 4% paraformaldehyde and stained with crystal violet solution for observation of colony formation.

### Metaphase Spread Analysis

Cells treated with DMSO or LPV for 5 days were exposed to APH (0.3 µm, 16 h), HU (4 mм, 4 h), or HU (4 mм, 4 h)+mirin (50 µm, 4 h), incubated with colcemid (0.1 µg mL^−1^) for 4 h, detached with trypsin and collected in 15‐mL Eppendorf tubes. Afterward, a hypotonic solution of KCl (0.075 м) was added, and the tubes were incubated at 37 °C for 20 min. The cells were then fixed with freshly prepared Carnoy's solution (methanol/acetic acid, 3:1). The prepared samples were added dropwise onto clean oil‐free slides from a height of 10 cm and stained with modified Giemsa staining solution (Beyotime, C0131). The chromosome structure was observed after mounting with neutral balsam.

### Cell Synchronization

To synchronize cells in the G1 phase, the cells were grown for 48 h in a medium containing 0.5% FBS. To synchronize cells in the S phase, cells were treated with APH (1 µm) overnight, and the medium was then changed to an APH‐free medium for another 2 h. For M phase synchronization, the cells were treated with nocodazole (150 ng mL^−1^) for 14 h, and the medium was then changed to a medium without nocodazole for another hour of culture.

### Isolation of Chromatin‐Bound Proteins

Cells in 10‐cm dishes were collected and resuspended in ice‐cold buffer A (150 mм NaCl, 50 mм HEPES (pH 7.5), 1 mм EDTA, protease inhibitor cocktail, and phosphatase inhibitor cocktail) containing 0.1% Triton X‐100 and incubated for 3 min. The supernatant (Dt fraction) was collected after centrifugation at 13 000 rpm for 3 min. The precipitate was washed twice with buffer A without Triton X‐100 and was then resuspended and incubated in buffer B (150 mм NaCl, 50 mм HEPES 7.5 (pH 7.5), 1 mм EDTA, 200 µg mL^−1^ RNase A, protease inhibitor cocktail, and phosphatase inhibitor cocktail) at RT for 30 min. The Rn fraction (supernatant) and Chr fraction (precipitate) were obtained by centrifugation. The subcellular fractions were then subjected to western blot analysis.

### Dual Luciferase Reporter Gene Assay

The promoter fragments (≈1 kb) of *FANCD2* and *BRCA1* containing the E2F elements were amplified and inserted into the pGL3‐basic vector upstream of the firefly luciferase sequence. The primers for firefly luciferase expression constructs are provided below:

*FANCD2*‐forward 5′‐CACCCTAGGAAGGGAAATGATA‐3′

*FANCD2*‐ reverse 5′‐CTACGACCATTGCTCCACTTAC‐3′

*BRCA1*‐forward 5′‐ACGTTGTCACCTCGCATTC‐3′

*BRCA1*‐reverse 5′‐GAAACCCCACAGCCTGTC‐3′

Next, the constructed firefly luciferase reporter vector (1 µg) and the *Renilla* luciferase reporter vector pRL‐TK (100 ng) were cotransfected into MCF7 cells with either E2F1‐3 × flag‐pCDNA3.1 (1 µg) or 3 × flag‐pCDNA3.1 (1 µg). Untransfected cells were used as controls. Three days later, according to the instructions of the dual luciferase reporter detection kit (Yeasen, 11402ES60), freshly prepared firefly luciferase reaction solution (100 µL) and *Renilla* luciferase reaction solution (100 µL) were added sequentially to cell lysate (20 µL). For each reaction, the luminescence intensity was measured, and the expression fold change was calculated.

### Coimmunoprecipitation

Protein A/G magnetic beads were washed three times with PBST (pH 7.4, 0.1% Tween 20) before incubation with the antibody diluted in PBST at 4 °C overnight. The bead‐antibody complexes were then washed three times with TBST (pH 7.4, 0.3% Triton X‐100) to remove the unbound fraction. Cell lysates were added to the bead‐antibody complexes, and the mixtures were incubated overnight at 4 °C to form bead‐antibody‐antigen complexes. The bead‐antibody‐antigen complexes were then washed 5–6 times with TBST, leaving a small amount of wash buffer for the last wash. The remaining solution was mixed with an equal volume of 2 × SDS loading buffer and boiled at 95 to 100 °C for 5 min. Finally, protein interactions were verified by SDS‒PAGE.

### Statistical Analysis

Unpaired Student's *t*‐tests were used to compare two groups of normally distributed data, while the Mann–Whitney test was used to analyze differences between two groups of nonnormally distributed data. For comparisons between multiple groups of data, a one‐way analysis of variance (ANOVA) was performed. The specific application of these significant difference tests can be found in the figure legends. All differential analyses were evaluated using GraphPad Prism 9. The difference between groups was determined according to the *p*‐value. ^*^ *p* < 0.05, ^**^ *p* < 0.01, ^***^ *p* < 0.001, ^****^ *p* < 0.0001. Quantitative analysis results are shown as the mean ± SD.

## Conflict of Interest

The authors declare no conflict of interest.

## Author Contributions

S.C. and L.W. designed the research. P.N. and C.Z. performed the experiments. P.N., C.Z., F.W., S.C., and L.W. analyzed the data. P.N., C.Z., S.C., and L.W. wrote and revised the manuscript. All the authors provided constructive comments on the manuscript and approved the final manuscript.

## Supporting information

Supporting Information

## Data Availability

The data that support the findings of this study are available from the corresponding author upon reasonable request.
