# Supplementary material for: The Compromised Fanconi Anemia Pathway in Prelamin A‐Expressing Cells Contributes to Replication Stress‐Induced Genomic Instability
Source: Adv Sci (Weinh). 2024 Jun 18;11(30):2307751. doi: 10.1002/advs.202307751 (PMC11321653; doi:10.1002/advs.202307751)
Supplement: Supplementary file 1 — Supporting Information [file ADVS-11-2307751-s001.docx]

Supporting Information

**The Compromised Fanconi Anemia Pathway in Prelamin A-Expressing Cells Contributes to Replication Stress-Induced Genomic Instability**

*Pengqing Nie, Cheng Zhang, Fengyi Wu, Shi Chen,** *and Lianrong Wang**


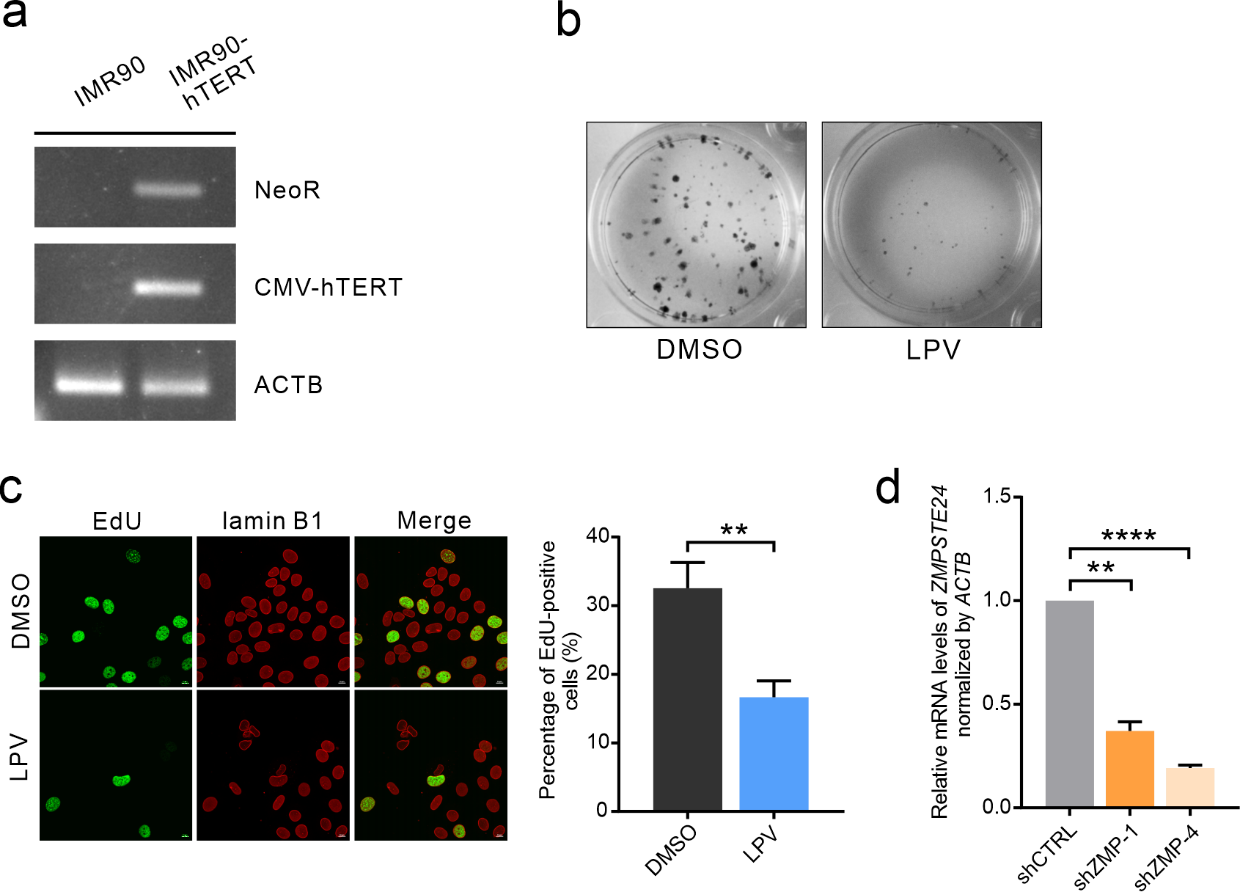


**Figure S1.**

Proliferation defects in prelamin A-expressing cells. a) The *NeoR* and *hTERT* genes were amplified by PCR from the genome of IMR90 fibroblasts stably expressing hTERT (IMR90-hTERT) and were detected by agarose gel electrophoresis. b) Crystal violet staining was used to visualize the colonies formed by cells grown for two weeks in media supplemented with DMSO or LPV (20 μм). c) Immunofluorescence staining was used to detect EdU incorporation after LPV (20 μм, 6 days) treatment. Scale bars: 10 μm. The percentage of EdU-positive cells was calculated. *n*=3. d) The silencing efficiency of the shRNA targeting *ZMPSTE24* was measured by qPCR. *n*=3. Quantitative analysis results are shown as the mean ± SD. *P* values were determined by unpaired Student’s *t* test. ***p* < 0.01; *****p* < 0.0001.


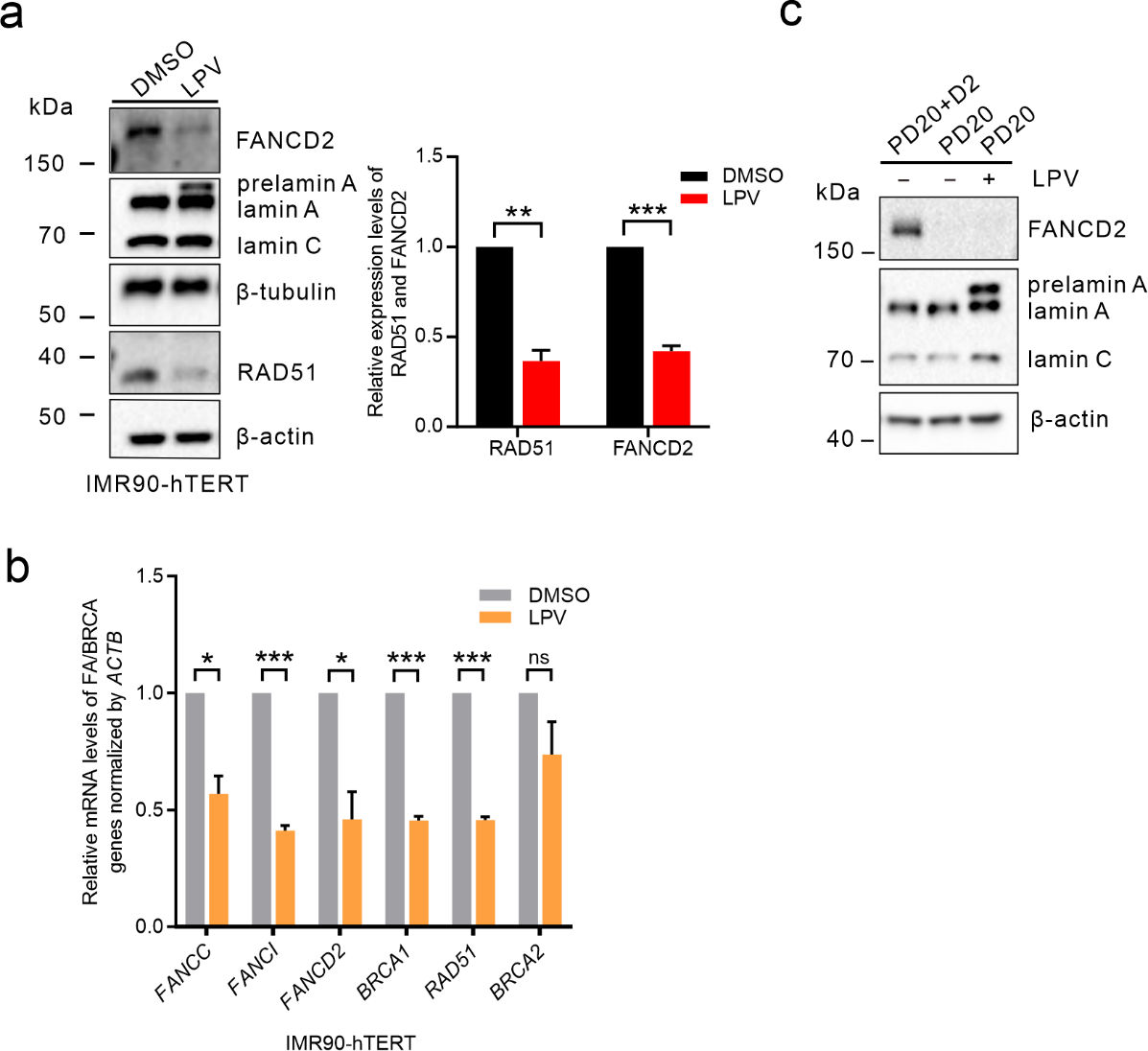


**Figure S2.**

Downregulation of the FA/BRCA gene network in prelamin A-expressing cells. a) The expression of FANCD2 and RAD51 in DMSO- and LPV (20 μм, 6 d)-treated IMR90-hTERT fibroblasts was measured by western blotting. *n*=3. b) mRNA levels of FA/BRCA genes in LPV (20 μм, 6 d)-treated IMR90-hTERT fibroblasts were measured by qPCR. *n*=3. c) The expression of prelamin A was detected in PD20 and PD20+D2 cells. Quantitative analysis results are shown as the mean ± SD. *P* values were determined by unpaired Student’s *t* test. ns, not significant; **p* < 0.05; **p < 0.01; ***p < 0.001.


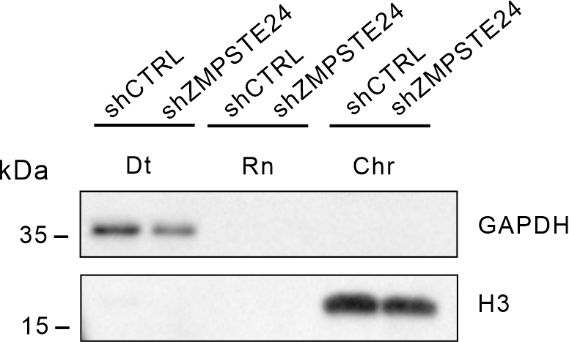


**Figure S3.**

Evaluation of the purity of the extracted chromatin-bound protein fraction. The purity of the extracted chromatin-bound protein fraction was evaluated by western blotting. Dt, detergent-extractable portion; Rn, RNase-extractable portion; Chr, chromatin portion.


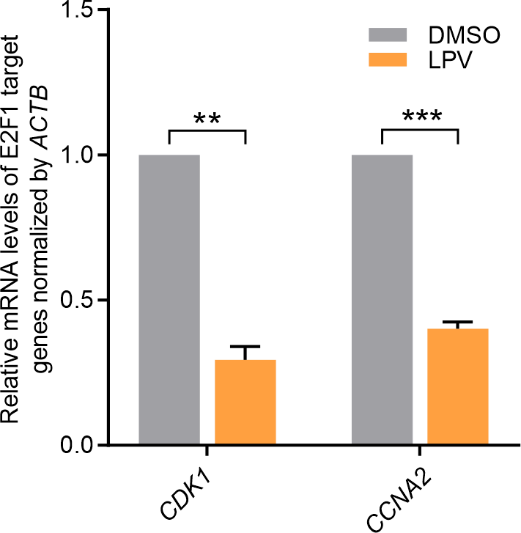


**Figure S4.**

Downregulation of *CDK1* and *CCNA2* in prelamin A-expressing cells. mRNA levels of *CDK1* and *CCNA2* in LPV (20 μм, 6 d)-treated IMR90-hTERT fibroblasts were measured by qPCR. *n*=3. Quantitative analysis results are shown as the mean ± SD. *P* values were determined by unpaired Student’s *t* test. ***p* < 0.01; ****p* < 0.001.


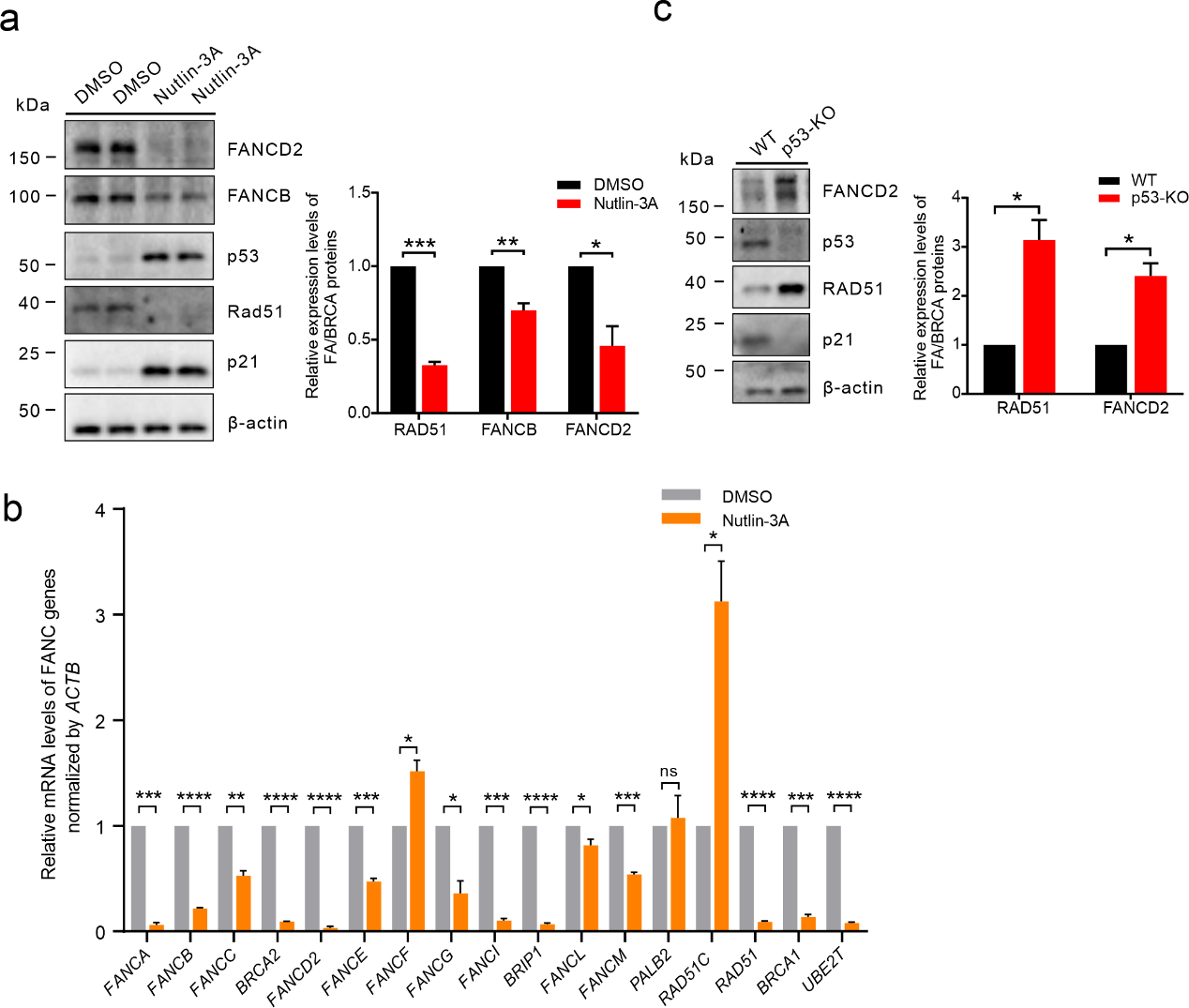


**Figure S5.**

p53 downregulates FA/BRCA genes. a) The expression of FANCD2, FANCB, and RAD51 in DMSO- and Nutlin-3A (10 μм, 24 h)-treated IMR90-hTERT fibroblasts was measured by western blotting. *n*=3. b) mRNA levels of FA/BRCA genes in DMSO- and Nutlin-3A (10 μм, 24 h)-treated IMR90-hTERT fibroblasts were measured by qPCR. *n*=3. c) The expression of FANCD2 and RAD51 in p53-knockout IMR90-hTERT fibroblasts was measured by western blotting. *n*=3. Quantitative analysis results are shown as the mean ± SD. *P* values were determined by unpaired Student’s *t* test. ns, not significant, **p* < 0.05; ***p* < 0.01, ****p* < 0.001, *****p* < 0.0001.

**Table S1.** Primers used to validate IMR90-hTERT fibroblasts.

| **Gene** | **Forward Primer (5’-3’)** | **Reverse Primer (5’-3’)** |
| --- | --- | --- |
| NeoR | ATGGATTGCACGCAGGTTCTC | TCGGCAGGAGCAAGGTGAGA |
| CMV-TERT | GCAAATGGGCGGTAGGCGT | CGTCCCAGGGCACGCACA |
| ACTB | CATCGTCCACCGCAAATGCTT | GGGACTTCCTGTAACAACGCATC |

**Table S2.** shRNA, siRNA and gRNA targeting sequences.

| **Name** | **Sequence (5’-3’)** |
| --- | --- |
| shCTRL | CCTAAGGTTAAGTCGCCCTCG |
| shZMPSTE24-1 | CGACTCTATCAACTGGATAAA |
| shZMPSTE24-4 | CCCTGTTTCTGACTGGTTGTT |
| siE2F1 | GUGAUUUAUUUAUUGGGAA |
| siFANCD2 | CAACAUACCUCGACUCAUU |
| siNC | RIBOBIO, siN0000001-1-5 |
| p53-gRNA | CCATTGTTCAATATCGTCCG |

**Table S3.** qPCR primers.

| **Gene** | **Forward Primer (5’-3’)** | **Reverse Primer (5’-3’)** |
| --- | --- | --- |
| *FANCA* | GCGTGTACCATTCTTGTCAAC | GCTACCATCTCCTGCAATCTG |
| *FANCB* | CAAATCCTTTCCCAGCACCAT | GACCCTTTTTGCTTCCAATCC |
| *FANCC* | TGTAAACGAGGCCATTTTGC | TTTTTCAAGGCTGGGAAGGT |
| *BRCA2* | ACCATATTTACCATCACGTGCACTA | TGCACCATCTTGCAAAGCA |
| *FANCD2* | AGACTGTCAAAATCTGAGGATAAAGAGA | TGGTTGCTTCCTGGTTTTGG |
| *FANCE* | TCTGGATGATGCTAAAGGTCTGG | TGAAGAAGCTGTAGCTCAACTG |
| *FANCF* | CCCCTCCACCTCTGAAAGAT | CTCCATCCTGCGCTTTACA |
| *FANCG* | GACAGCAGTTGGCTCAGGAT | CAGTCAGCTCCAAGGGAAGA |
| *FANCI* | CAGGCAACCCTACCAAATCAG | GCAGAGTTCCCAGTTGCATGA |
| *BRIP1* | AGAGACTTGGGCTCTACACCTG | GTGTGGTGATTGCCAATATGA |
| *FANCL* | GGAGTTGAAGATGCTTTTGGA | TCAATAAGGCTTGAGTAGAACTGG |
| *FANCM* | TGGAGACTGTGGCAAGATCATC | GCGGCATCGATCTGAGTGA |
| *PALB2* | CCGGTTGTAAAGAGCCATGTATC | ATCCAGAGCTTTCCAAAGAGAAAC |
| *RAD51C* | GTGGCAGGTGAAGCAGTTTTTA | GCAAGGTCTACCACTCTATCAACCA |
| *RAD51* | TGGGAGATGCCAAAGACTGAA | AGGCTGCAGCACTTAAGGTTTT |
| *BRCA1* | TTCAGGCTGTTGTTGGCTTA | GCTATTCTCTTGAGGCCAAGC |
| *UBE2T* | GGGATCATGCAGAGAGCTTCA | GGGTGGCTCTGTGGCTAACA |
| *ZMPSTE24* | TTATGATAGCCAACCCACT | TTTCTTGGCAAATGCATC |
| *p53* | AGGCCTTGGAACTCAAGGAT | CCCTTTTTGGACTTCAGGTG |
| *p21* | AGACCATGTGGACCTGTCACTG | GTTTGGAGTGGTAGAAATCTGTC |
| *CCNA2* | CACTCTACACAGTCACGGGA | AGTGTCTCTGGTGGGTTGAG |
| *CDK1* | GGATGTGCTTATGCAGGATTCC | CATGTACTGACCAGGAGGGATAG |
| *ACTB* | CGTCACCAACTGGGACGACA | CTTCTCGCGGTTGGCCTTGG |
